# Rural-urban correlates of modern contraceptives utilization among adolescents in Zambia: a national cross-sectional survey

**DOI:** 10.1186/s12905-022-01914-8

**Published:** 2022-08-02

**Authors:** Quraish Sserwanja, Milton W. Musaba, Linet M. Mutisya, David Mukunya

**Affiliations:** 1 Programs Department, GOAL, Arkaweet Block 65 House No. 227, Khartoum, Sudan; 2 Department of Obstetrics and Gynaecology, Busitema University, Mbale, Uganda; 3 Maternal and Child Health Project, Swedish Organization for Global Health, Mayuge, Uganda; 4 Department of Public Health, Busitema University, Mbale, Uganda; 5 Sanyu Africa Research Institute, Mbale, Uganda

## Abstract

**Background:**

Modern contraceptive use among adolescents is low despite the adverse effects of adolescent pregnancies. Understanding correlates of modern contraceptive use in different settings is key to the design of effective context-specific interventions. We aimed to determine factors associated with modern contraceptives use among adolescents in rural and urban settings of Zambia.

**Methods:**

We analyzed secondary data from 2018 Zambia demographic and health survey (ZDHS) focusing on adolescent girls aged 15–19 years. We used multivariable logistic regression in SPSS version 25 to examine rural-urban variations in factors associated with modern contraceptive utilization.

**Results:**

Overall, 12.0% (360/3000, 95% CI: 10.9–13.2) of adolescents in Zambia were using modern contraceptives. Use of modern contraceptives was higher in rural areas at 13.7% (230/1677, 95% CI: 12.1–15.3) compared to 9.8% (130/1323, 95% CI: 8.3–11.6) in urban areas. In the rural areas, having a child (aOR = 13.99; 95% CI 8.60–22.77), being married (aOR = 2.13; 95% CI 1.42–3.18), being older at 19 years (aOR = 3.90; 95% CI 1.52–10.03), having been visited by a field health worker (aOR = 1.62; 95% CI 1.01–2.64), having been exposed to family planning messages on mass media (aOR = 2.87; 95% CI 1.01–8.18) and belonging to the richest wealth quintile (aOR = 2.27; 95% CI 1.43–3.62) were associated with higher odds of contraceptive utilization. Furthermore, adolescents in the Northern (aOR = 0.29; 95% CI 0.11–0.80) and Luapula (aOR = 0.35; 95% CI 0.15–0.81) provinces were associated with less odds of utilizing contraceptives compared to those in Western province. In the urban areas, older age at 19 years (aOR = 4.80; 95% CI 1.55–14.84) and having a child (aOR = 18.52; 95% CI 9.50–36.14) were the only factors significantly associated with modern contraceptive utilization.

**Conclusion:**

Age and having a child were associated with modern contraceptive use in both rural and urban areas. In rural areas (province, marital status, being visited by field health workers, family planning messages exposure and wealth index) were the only associated factors. This indicates that interventions aiming to increase contraceptive utilization should be context specific.

**Supplementary Information:**

The online version contains supplementary material available at 10.1186/s12905-022-01914-8.

## Background

Globally, adolescent fertility rate is high with their birth rate standing at 44 births per 1000 women [1]. Africa faces the highest burden of adolescent fertility with sub-Saharan Africa standing at 101 births per 1000 females [1, 2]. Not using modern contraceptive method contributes to over 90% of the annual unplanned pregnancies among adolescents in sub-Saharan Africa, Latin America and the Caribbean, and South Central and Southeast Asia [1].

Adolescent childbearing is associated with adverse health risks such as; increased maternal mortality and morbidity such as obstetric fistulae and sexually transmitted infections [3, 4]. Furthermore, babies born to adolescent mothers are at an increased risk of perinatal mortality because of low birth weight and problems of prematurity [3, 5]. Despite the adverse effects of adolescent pregnancies and births, contraceptive use among adolescents in Africa is low [3, 6, 7]. In addition to the negative health outcomes, adolescent childbirths and sexually transmitted infections (STIs) have been associated with adverse socio-economic consequences such as; poverty, school dropout and subsequent lower educational attainment which affects their personal development [1, 2, 8, 9].

Promoting use of modern contraceptives in this age group is a priority intervention that will ensure improvement of pregnancy related outcomes [10, 11]. Due to the heterogeneous nature of the sub-Saharan region, sub-regional variations exist in contraceptive use among young women [12]. Despite the various interventions and policies to promote modern contraceptives utilization among young women in Sub-Saharan Africa, the rate has remained low [3, 13, 14]. This could partly be attributed to generic interventions and policies that are not adapted to local settings [13].

Zambia’s adolescent contraceptive use is low despite high universal knowledge of at least one modern contraceptive method [3]. The government of Zambia introduced youth friendly health services (YFHS) in 1994 in selected facilities of Eastern, Lusaka, Southern and Copperbelt provinces. However, a mid-term review of this programme showed lack of standards of care for adolescents, poorly coordinated service delivery and limited access to care (national standards and guidelines for adolescent friendly health services). This further led to the national adolescent health strategic plans (NAHSP) (2011–2015, 2017–2021) that aimed for appropriate, accessible, efficient and effective adolescent friendly health services (ADFHS) [15, 16]. The government introduced strategies such as increasing access to ADFHS, strengthening youth involvement in the planning, implementation, evaluation and monitoring of ADFHS, strengthening the procurement and distribution of adolescent health essential medical products including contraceptives [15, 16]. Mercy et al. evaluated the impact of the NAHSP on adolescent sexual reproductive health services in Zambia noted successes such as consistence and standardized packages for adolescent health, evidence based decision making and policy harmonization [17]. However, gaps such as inadequate human resources, infrastructure hence limited space for privacy, unclear policy on age of consent with those under 16 years being asked for parental consent to access contraceptives and limited funding have negatively affected service delivery and access to adolescent friendly care [17].

Inequalities in access to adolescent friendly health services including contraceptives have been documented with significant urban-rural differences existing not only in Zambia but also in sub-Saharan Africa (SSA) [3, 12]. Furthermore, inequalities in terms of adolescent related health challenges have been documented with a more recent publication showing rural Zambian adolescents contributing to 71% of the teenage pregnancy compared to 29% of urban adolescents [2]. In the NAHSP impact assessment, Mercy et al. recommended the ministry of health to change on how the health system responds to adolescent health needs. Context specific (rural-urban) policies and strategies could be crucial to ensure that the needed change on how the health system responds to the health needs of adolescents is effective and efficient. Information on the predictors of the rural-urban differences in the uptake of modern contraceptive utilization among female adolescents is relevant since policies, programs, and interventions that are necessary to improve modern contraceptive uptake may differ in rural and urban areas. Although disparities in the rates of utilization of contraceptives between urban and rural adolescents have been documented [3, 18], information on factors associated with these differences has not been adequately explained. It is important to further understand these factors when stratifying by rural-urban place of residence among adolescents. Understanding the various associated factors of modern contraceptive uptake in different settings is key to designing effective context-specific interventions tailored to the needs of each setting. Therefore, we aimed to determine the factors associated with modern contraceptive utilization among adolescents in Zambia stratified by rural-urban place of residence.

## Materials and methods

### Study data

We conducted a secondary data analysis using the 2018 Zambia Demographic and Health Survey (ZDHS) dataset after obtaining permission from the MEASURE DHS project. Demographic health surveys (DHS) are national surveys that are conducted periodically in low and middle income countries (LMICs) standardized to enable comparisons among the different countries [19, 20]. The 2018 ZDHS is the sixth in a series of demographic and health Surveys with the previous surveys conducted in 1992, 1996, 2001–02, 2007, and 2013–14 [19]. The 2018 ZDHS employed a two-stage stratified sampling method which yielded 13,625 households using the 2010 Zambia’s Census of Population and Housing (CPH) sampling frame [19]. The 2018 ZDHS data were collected between 18th July 2018 and 24th January 2019 [19]. The ZDHS is designed to provide up-to-date information on health indicators using household, women’s, men’s and biomarker questionnaires [19]. The data used in this study was collected using the women’s questionnaire. This analysis included only adolescents aged 15–19 years whose total sample size was 3000 out of the total women sample of 13,683 of which 10,683 were excluded since they were aged 20–49 years [19]. Female adolescents were asked if they were currently using any method of modern contraception [19].

### Study sampling and participants

The 2018 ZDHS was implemented by the Zambia Statistics Agency in partnership with the ministry of health; the University Teaching Hospital Virology Laboratory (UTH-VL); and the department of population studies at the University of Zambia (UNZA) [19]. The ZDHS used two-stage systematic sampling to select participants from households nested in clusters (enumeration areas) across the all the provinces of Zambia [19]. Women aged 15–49 years who were either permanent residents or visitors who had stayed in the selected households the night before the survey were eligible for interviews with a total of 13,683 women being interviewed [19]. Of these, a weighted sample of 3,000 (1677 rural and 1323 urban) were adolescents aged between 15 and 19 years who had consented and were interviewed. Written informed consent was provided by all participants of the survey. Written permission to access the whole ZDHS database was obtained through DHS program [21]. Flow diagram for the sampling process is included in Additional file 1: Fig. S1. A detailed explanation of the sampling process is available in the ZDHS 2018 report [19].

### Outcome variables

Utilization of any method of modern contraceptive method (sterilization, injectable, intrauterine devices (IUDs), contraceptive pills, implants, condoms, the standard days method, lactational amenorrhea, and emergency contraception) [19] was coded as one (1) while non-utilization was coded as zero (0).

### Independent variables

This study included determinants of modern contraceptives utilization basing on evidence from available literature and data [4, 22, 23]. We included twelve explanatory variables in the analysis. Wealth index is a measure of relative household economic status and was calculated by DHS from information on household asset ownership using Principal Component [19]. Independent variables were categorized into;

### Individual characteristics

Level of education (no education, primary and post primary), age (15, 16, 17, 18 and 19), marital status (married and not married), has a child or children (yes and no), working status (working and not working), exposure to family planning messages on television (TV), radio, mobile phone and newspapers as a composite variable (yes and no), being visited by a field health worker (yes and no), difficulty in seeking permission for getting healthcare (big problem and no big problem) and problems with distance to nearest health facility (big problem and no big problem).

### Household characteristics

Wealth index of household (categorized into quintiles: richest, richer, middle poorer and poorest), province of residence (all the 10 provinces of Zambia), and sex of household head (female and male).

### Statistical analysis

To account for the unequal probability sampling in different strata [24] and to ensure representativeness of the survey results at all levels, we used sample weights [25]. Furthermore, to account for the multi-stage cluster study design, complex sample analysis was performed using SPSS version 25.0 statistical software. Proportions were tabulated for each of the categorical independent variable. Bivariable logistic regression was done to the association of each exposure with the outcome variable and we presented crude odds ratio (COR), 95% confidence interval (CI) and *p*-values. Independent variables found significant at bivariable level (*p*-value < 0.25) were included in the final multivariable logistic regression model. Two separate models for urban and rural samples were fitted with the first model (Rural Model) fitted to identify the predictors of modern contraceptive utilization for rural adolescents and the second model (Urban Model) to identify predictors among urban adolescents. The independent variables used for both rural and urban adolescents were the same to enable a comparison between both areas. Adjusted odds ratios (AOR), 95% Confidence Intervals (CI) and *p*-values were calculated with statistical significance level set at *p*-value < 0.05. All variables in the model were assessed for collinearity, however, none of the assessment among both rural and urban variables had a variance inflation factor (VIF) greater than 3.

## Results

Table 1 shows a comparison of selected background characteristics of female adolescents in rural and urban Zambia. Rural areas had more participants (1677) compared to urban areas (1323). Remarkable differences were observed in exposure to family planning messages on mass media with 11.5% in rural areas being exposed compared to 23.8% in urban areas. Furthermore, 22.5% of rural adolescents were working compared to 11% in urban areas, 21.1% in rural areas were married compared to 6.3% in urban areas and 38.9% had post-primary education in rural areas compared to 73% in urban areas. Almost half (48.3%) of the adolescents in urban areas belonged to the richest wealth quintile compared to 4.2% in rural areas. Big problems with distance to nearest health facility were higher in rural areas at 39.3% compared to 11.8% in urban areas. More detailed characteristics of study participants are shown in Table 1. Overall, 12.0% (360/3000, 95% CI: 10.9–13.2) of the female adolescents in Zambia were utilizing modern contraceptives. Utilization of modern contraceptives was higher in rural areas at 13.7% (230/1677, 95% CI: 12.1–15.3) compared to 9.8% (130/1323, 95% CI: 8.3–11.6) in urban areas.

**Table 1:** Rural-Urban background characteristics of adolescents as per ZDHS 2018

Characteristics	Total *N* = 3000	%	Rural *N* = 1677	%	Urban *N* = 1323	%
*Household number*						
Less than 6	982	32.8	527	31.4	455	34.4
6 and above	2018	67.2	1150	68.6	868	65.6
*Sex of household head*						
Female	834	27.8	413	24.6	421	31.8
Male	2166	72.2	1264	75.4	902	68.2
*Provinces*						
Central	297	9.9	195	11.6	102	7.7
Copper belt	491	16.4	67	4.0	424	32.0
Eastern	342	11.4	295	17.6	47	3.5
Luapula	253	8.4	191	11.4	62	4.7
Lusaka	475	15.8	60	3.6	415	31.4
Muchinga	191	6.4	156	9.3	35	2.6
Northern	248	8.3	196	11.7	53	4.0
North Western	186	6.2	146	8.7	41	3.1
Southern	327	10.9	232	13.8	95	7.2
Western	190	6.3	139	8.3	51	3.8
*Exposure to FP messages*						
Yes	506	16.9	192	11.5	314	23.8
No	2494	83.1	1485	88.5	1009	76.2
*Working status*						
Not working	2477	82.6	1299	77.5	1178	89.0
Working	523	17.4	378	22.5	145	11.0
*Marital status*						
Not married	2563	85.4	1323	78.9	1240	93.7
Married	437	14.6	354	21.1	83	6.3
*Education level*						
No education	99	3.3	88	5.2	11	0.8
Primary education	1283	42.8	937	55.8	346	26.2
Post primary education	1618	53.9	653	38.9	965	73.0
*Wealth index*						
Poorest	510	17.0	502	29.9	8	0.6
Poorer	541	18.0	511	30.5	30	2.2
Middle	585	19.5	439	26.2	146	11.0
Richer	655	21.8	154	9.2	501	37.9
Richest	709	23.7	70	4.2	639	48.3
*Age*						
15	653	21.8	397	23.6	256	19.4
16	530	17.6	306	18.3	224	16.9
17	552	18.4	287	17.1	265	20.1
18	722	24.1	415	24.7	307	23.2
19	543	18.1	272	16.1	271	20.4
*Has a child or children*						
No	2277	75.8	1175	70.0	1102	83.3
Yes	723	24.2	502	30.0	221	16.7
*Visited by field health worker*						
Yes	244	8.1	152	9.1	92	6.9
No	2756	91.9	1525	90.9	1231	93.1
*Problems seeking permission for care*						
Big problem	112	3.7	93	5.5	19	1.4
Not big problem	2888	96.3	1584	94.5	1304	98.6
*Problems with distance to care*						
Big problem	815	27.2	659	39.3	156	11.8
Not big problem	2185	72.8	1018	60.7	1167	88.2
*Contraception use*						
Yes	360	12.0	230	13.7	130	9.8
No	2640	88.0	1447	86.3 55 5 66 3f 444 888	1193	90.2

*FP* Family planning

### Determinants of rural and urban utilization of modern contraceptives

Table 2 presents results of multivariable logistic regression analysis showing predictors of rural and urban utilization of contraceptives among rural and urban female adolescents. Our analysis revealed that older age (19 years) and history of childbirth were associated with higher odds of modern contraceptive utilization among adolescents from both regions of residence. In the rural areas, having a child (aOR = 13.99; 95% CI 8.60–22.77), being married (aOR = 2.13; 95% CI 1.42–3.18). being older at 19 years (aOR = 3.90; 95% CI 1.52–10.03), having been visited by a field health worker (aOR = 1.62; 95% CI 1.01–2.64), having been exposed to family planning messages on mass media (aOR = 2.87; 95% CI 1.01–8.18) and belonging to the richest wealth quintile (aOR = 2.27; 95% CI 1.43–3.62) were associated with higher odds of contraceptive utilization. Furthermore, adolescents in the Northern (aOR = 0.29; 95% CI 0.11–0.80) and Luapula (aOR = 0.35; 95% CI 0.15–0.81) provinces were associated with less odds of utilizing contraceptives compared to those in Western province.

In the urban areas, older age at 19 years (aOR = 4.80; 95% CI 1.55–14.84) and having a child (aOR = 18.52; 95% CI 9.50–36.14) were the only factors significantly associated with modern contraceptive utilization.

**Table 2:** Determinants of rural urban utilization of contraceptives among female adolescents in Zambia

Characteristics	Rural COR (95%CI)	*p*-value	Rural AOR (95%CI)	Urban COR (95%CI)	*p*-value	Urban AOR (95%CI)
Sex of family head		0.356			0.078	
Male	1			1		1
Female	0.83 (0.56–1.24)			0.60 (0.34–1.06)		0.59(0.28–1.22)
Provinces		< 0.001			0.882	
Western	1		1	1		1
Copperbelt	**0.43 (0.19–0.99)**		0.57 (0.21–1.58)	0.87 (0.32–2.34)		-
Eastern	0.89 (0.49–1.62)		0.69 (0.30–1.58)	0.71 (0.17-3.00)		-
Luapula	**0.37 (0.19–0.74)**		**0.35 (0.15–0.81)**	0.54 (0.17–1.73)		-
Lusaka	0.71 (0.31–1.62)		0.87 (0.35–2.19)	0.76 (0.26–2.20)		-
Muchinga	0.51 (0.25–1.04)		0.45 (0.18–1.13)	0.90 (0.20–3.99)		-
Northern	**0.28 (0.12–0.65)**		**0.29 (0.11–0.80)**	0.55 (0.14–2.23)		-
North Western	1.39 (0.76–2.56)		2.00 (0.80–5.01)	1.16 (0.32–4.25)		-
Southern	0.57 (0.29–1.14)		0.57 (0.22–1.49)	0.48 (0.15–1.55)		-
Central	0.71 (0.34–1.50)		0.75 (0.30–1.91)	0.92 (0.33–2.56)		-
FP messages exposure		0.035			0.286	
No	1		1	1		
Yes	**1.55 (1.03–2.33)**		**2.27 (1.43–3.62)**	1.39 (0.76–2.53)		
Working status		< 0.001			0.029	
Not working	1		1	1		1
Working	**1.89 (1.39–2.56)**		0.95 (0.67–1.36)	**2.13 (1.08–4.21)**		0.76 (0.24–2.43)
Marital status		< 0.001			< 0.001	
Not Married	1		**1**	1		1
Married	**7.32(5.26–10.18)**		**2.13 (1.42–3.18)**	**8.19(4.06–16.53)**		1.28 (0.58–2.80)
Education level		0.271			0.262	
No Education	1			1		
Primary Education	1.49 (0.70–3.14)			3.44 (0.49–24.02)		
Post Primary Education	1.22 (0.56–2.62)			2.45 (0.36–16.83)		
Wealth index		0.061			0.010	
Poorest	1		1	1		1
Poorer	0.89 (0.63–1.26)		1.15 (0.72–1.85)	0.73 (0.14–3.99)		0.21 (0.01–3.85)
Middle	**0.63 (0.42–0.95)**		0.97 (0.57–1.67)	1.44 (0.24–8.75)		0.61(0.03–14.11)
Richer	**0.47 (0.23–0.97)**		0.67 (0.32–1.40)	1.35 (0.22–8.19)		1.09(0.04–26.85)
Richest	0.48 (0.20–1.17)		**2.87 (1.01–8.18)**	0.49 (0.07–3.08)		1.07(0.03–29.54)
Age		< 0.001			< 0.001	
15 16	1 2.69 (0.97–7.41)		1 1.85 (0.67–5.13)	1 0.54 (0.18–2.11)		1 0.29 (0.07–1.29)
17	**5.34 (2.11–13.56)**		2.08 (0.80–5.42)	**4.43 (1.23–15.94)**		1.65 (0.37–7.40)
18	**9.83 (3.94–24.49)**		2.46 (0.95–6.38)	**6.84 (2.48–18.88)**		1.84 (0.62–5.46)
19	**20.28 (8.38–49.08)**		**3.90 (1.52–10.03)**	**17.61(7.02–44.11)**		**4.80(1.55–14.84)**
Gave birth		< 0.001			< 0.001	
No	1		1	1		1
Yes	**23.13(14.83–36.07)**		**13.99(8.60-22.77**	**25.85(13.13–50.88**		**18.52(9.50-36.14**
Field work visited		0.001			0.727	
No	1		1	1		
Yes	**2.09 (1.38–3.16)**		**1.62 (1.01–2.64)**	0.85 (0.34–2.14)		
Permission for care		0.820			0.516	
Big problem	1			1		
Not big problem	1.07 (0.59–1.95)			1.63 (0.37–7.10)		
Distance to care		0.654			0.460	
Big problem	1			1		
Not big problem	1.06 (0.81–1.40)			1.31 (0.64–2.65)		

Bold** =** Significant at *p*-value < 0.05

## Discussion

The utilization of modern contraceptives was higher in rural areas at 13.7% (230/1,677, 95% CI: 12.1–15.3) compared to 9.8% (130/1,323, 95% CI: 8.3–11.6) in urban areas. This could partly be attributed to the fact that rural areas had more married (21.1%) and had fewer adolescents with no child or children (70%) compared to urban areas where 6.3% of adolescents are married and 83.3% have never had a child.

Our study showed that contraceptive utilization in Zambia is lower than that of the sub-Saharan region [12], higher than that of Uganda [4], Chad and Benin [12]. The higher prevalence in Zambia could be partly attributed to the adolescents’ targeting evidence based strategies and policies in the NAHSPs that have been revised over the decade aimed at ADFHS [15, 17], the higher literacy rates observed in most southern Africa countries including Zambia, suggesting that these adolescents are more likely to have better capacity in areas of life such as socio-cultural and familial empowerment hence more likely to challenge the negative norms and beliefs, increase health literacy and adopt better health seeking behaviours [12].

The current study showed that age and having a child were common determinants of modern contraceptive use in both rural and urban settings. On the other hand, marital status, wealth index, being visited by field health worker, exposure to family planning messages and province were determinants of modern contraceptives use in rural areas but not in urban areas.

Older age (19 years) was associated with modern contraceptive utilization among both rural and urban adolescents compared to younger age (15 years). This association was stronger among urban adolescents compared to their rural counterparts. This finding is not surprising because Older age has been shown to be associated with modern contraceptive utilization in similar studies [3, 7, 27]. Older adolescents tend to be more exposed, knowledgeable and enlightened regarding availability and importance of contraceptives [7]. In addition, older adolescents are more likely to have had children, be married, and have obtained higher levels of education compared to their younger counterparts which factors have been documented to be positively associated with contraceptive utilization [7]. Furthermore, older age is associated with independence and better ability to make positive decisions about their health including contraception [1].

Having had a child was associated with higher odds of modern contraceptive utilization among both rural and urban adolescents compared to not having had a child. This study found that adolescents who had a child were more than 13 (in rural areas) and 18 (in urban areas) times more likely to practice contraceptive use compared to their counterparts who had no children. This might partly be attributed to improved health seeking behavioral and the desire to continue with school and not have repeat pregnancies [4, 26, 27]. Amongin et al. showed that after having the first child, adolescents show the need to delay the next pregnancy, highlighting their need to be supported with contraception [27]. Furthermore, adolescents that had ever given birth to a child were more likely to utilize health facilities for antenatal care, delivery and post-natal care services [27, 28] hence increased exposure to family planning counselling [4]. Childbirth has been documented to be associated with modern contraceptive utilization in similar studies [4, 26].

Marital status showed a significant association with modern contraceptives use among rural female adolescents. However, this was not observed among urban adolescents probably due to lack of power to detect this association that resulted from having very few married urban girls. Married or cohabiting adolescents had twice the odds of using contraceptives compared to their non-married counterparts. This could be attributed to better financial and social support among married adolescents from their partners and in laws hence easier affordability and access to contraceptives compared to their unmarried counterparts [4, 7]. Furthermore, married adolescents have regular exposure to sexual intercourse and the risk of unintended pregnancy [3]. Marital status has been shown to be associated with modern contraceptive use in similar contexts [3, 4, 7, 29].

Rural adolescents in the richest wealth quintile had thrice the odds of using contraception compared to their counterparts in the poorest quintile. This is consistent with findings from similar contexts [1, 4, 30]. Adolescents belonging to the poorest quintile are more likely to have limited access to modern contraceptives due to the direct and indirect costs incurred when accessing contraceptives [26, 31]. The poor are also more likely to be less informed about contraception due to the low education levels, less exposure to mass media which limits their health literacy and ability to make informed decisions [32–35]. Wealth has been shown to be associated with modern contraceptive use in other studies [26, 33, 36].

Province among rural adolescents was significantly associated with contraceptive utilization with adolescents in Luapula and Northern provinces having less odds of using contraceptives compared to those in the Western province. Luapula is among the provinces with the poorest health indicators such as the highest maternal mortality ratio attributable to the low quality of services, limited accessibility to care due to the associated costs worsened by the poor socio-economic situation in the province [37]. Furthermore health service delivery is affected by the limited numbers of health workers in these provinces with the Northern province having some of the highest doctor to population ratio and rural health centers’ vacancy rates [38]. The poor socio-economic status affects access to quality education, exposure to mass media which coupled with poor quality of health services impends utilization of contraceptives among adolescents. Regional differences have similarly been shown to be associated with contraceptive use in various studies [26, 39–41].

Exposure to family planning messages on mass media was associated with higher odds of modern contraceptive utilization among rural adolescents. Since most African parents rarely communicate about sexual and reproductive health (SRH) with their children, adolescents tend to resort to informal sources for SRH information [42]. Mass media especially mobile phones offer an anonymous, confidential and easily accessible space to find sensitive SRH information [42]. Mass media messages may promote modern contraceptives utilization through increased awareness, sensitization and debunking of myths hence positive health seeking behavior [43]. Besides being a source of SRH leading to increased contraceptive literacy, mass media can provide information on location of different contraceptive service providers and some like mobile phones can be used to purchase contraceptives [44, 45].

Unlike in urban areas, being visited by a field health worker among rural adolescents was associated with higher odds of modern contraceptive utilization. Shortage of health facilities and large distances needed to be covered by rural adolescents leading to a higher demand for community health workers compared to easier access of health facilities in urban areas could partly explain the observed difference in association [46–48]. These workforce shortage that are mainly experienced in rural Zambia affect timely access to quality health services [49]. Due to these challenges, about a decade ago, Community Health Assistants (CHAs) were introduced in through the National Community Health Worker Strategy to ensure easier access to health services mainly in rural Zambia [48]. In addition to their roles of providing basic primary health care services, CHAs ensure increased modern contraceptive utilization through health promotion and behavioral change sessions and providing basic reproductive, maternal and newborn health (RMNH) interventions were including contraceptive counselling and administration of injectable contraceptives within the communities [46, 48]. Being visited by field health workers has been shown to be associated with increased utilization of SRH services in several other studies [12, 50–52].

### Strengths and limitations

We used a nationally representative sample and weighed the data for analysis, so our results are generalizable to all Zambian female adolescents aged 15 to 19 years. Standardized procedures are a requirement of DHS surveys in data collection and validated questionnaires are used which ensures the internal and external validity of the results. However, use of cross-sectional data only enables associations to be established but not causal relationships.

### Conclusion and policy implications

This study demonstrates the considerable low prevalence of modern contraceptives utilization in rural and urban adolescents in Zambia using nationally representative data. Two factors (age and having a child) were found significant in both rural and urban areas while three (marriage, wealth index, being visited by field health workers, exposure to family planning mass media messages and province) were significant in only rural areas. This evidence suggests interventions aiming to increase contraceptive utilization should be context specific. Urban policies and programmes need to prioritize young adolescents who have never given birth. Strengthening SRH education sessions in schools could be key in accessing young adolescents who have never given birth. Rural SRH policies and projects need to target unmarried adolescents, those from the poorest households, from Northern and Luapula provinces and strengthening the work of CHAs. CHAs could be further supported through increasing their number, the supervision support system, capacity building and SRH medical supplies chain. Strengthening the inclusion of SRH workers in the production of SRH mass media content, supporting provision of newspapers/magazines containing SRH sections to adolescents in schools/ adolescent health units in health centres produced in easy to read and understand local languages could ensure increased coverage and effectiveness of mass media family planning messages.

## Supplementary Information

**Additional file 1: Fig. S1.** Flow chat of sampling process.

## Data Availability

The data set used is openly available upon permission from MEASURE DHS website (URL: https://www.dhsprogram.com/data/available-datasets.cfm).

## Abbreviations

AOR: Adjusted odds ratio
CI: Confidence interval
COR: Crude odds ratio
DHS: Demographic health survey
ZDHS: Zambia demographic health survey
OR: Odds ratio
SD: Standard deviation
WHO: World health organization
SPSS: Statistical package for social science
