# Supplementary material for: Rural-urban correlates of modern contraceptives utilization among adolescents in Zambia: a national cross-sectional survey
Source: BMC Womens Health. 2022 Aug 2;22:324. doi: 10.1186/s12905-022-01914-8 (PMC9344606; doi:10.1186/s12905-022-01914-8)
Supplement: Supplementary file 1 — Additional file 1: Fig. S1. Flow chat of sampling process. [file 12905_2022_1914_MOESM1_ESM.docx]

13,683 women aged 15 to 49 years

Excluded 10,683 women aged 20 to 49 years

3,000 adolescents aged 15 to 19 years

*Additional file Figure 1: flow chat of sampling process*
